# Treatment patterns in patients with newly diagnosed COPD in the USA

**DOI:** 10.1186/s12890-024-03194-4

**Published:** 2024-08-17

**Authors:** Antonio Anzueto, Sheri Rogers, Bonnie Donato, Beverly Jones, Kushal Modi, Abisola Olopoenia, Robert Wise

**Affiliations:** 1 South Texas Veterans Healthcare System, Division of Pulmonary Diseases/Critical Care Medicine, University of Texas Health, 111 E 7400 Merton Minter Blvd, San Antonio, TX 78229 USA; 2 Boehringer Ingelheim Inc, Ridgefield, CT USA; 3 Cerner Enviza, New York, NY USA; 4 Pulmonary and Critical Care Medicine, Johns Hopkins University School of Medicine, Baltimore, MD USA

**Keywords:** COPD, Exacerbations, Newly diagnosed, Treatment patterns, Maintenance therapy

## Abstract

**Background:**

Prompt and effective management with maintenance therapy (single or dual bronchodilator therapy) is recommended after the initial diagnosis of chronic obstructive pulmonary disease (COPD) to maintain lung function and prevent exacerbations. Contrary to guideline-based recommendations, most patients are not prescribed maintenance treatment at initial diagnosis. The current study assessed the pharmacologic treatment patterns and outcomes of newly diagnosed patients with COPD in the USA.

**Methods:**

This retrospective, noninterventional study used de-identified data from the Inovalon Insights’ database (Commercial, Medicaid Managed Care, and Medicare Advantage–insured individuals) between January 1, 2015, and December 31, 2021. The “patient journey” from initial diagnosis was followed over a 4-year period. The primary outcome measure was the number of moderate or severe exacerbations. Secondary outcome measures included the cumulative incidence of exacerbations, mean cumulative count of moderate and severe exacerbations, rates of moderate and severe exacerbations in patients who remained untreated after diagnosis in 12-month time periods for 4 years, sociodemographic and clinical characteristics, and pharmacologic treatment patterns.

**Results:**

The cohort consisted of 238,158 newly diagnosed patients with COPD (female [52.9%]; mean age 63.8 years). The majority of patients with COPD had Medicaid as their primary insurance (46.2%). Overall, during the 4-year follow-up period, 32.9% of the patients had at least one moderate or severe exacerbation, and 25.8% and 13.8% experienced moderate and severe exacerbations, respectively. At diagnosis, 86.2% of the patients were untreated and most remained untreated by the end of the follow-up (63.8%). Most patients (62.0%) received long-acting beta-agonist (LABA)/inhaled corticosteroids (ICS) as their initial treatment at diagnosis, and LABA/ICS continued to be the most common initial treatment during the 4-year period (64.0% at year 1; 58.0% at year 4).

**Conclusions:**

Most patients with COPD were not treated at initial diagnosis and remained untreated during follow-up. Our data highlight a lack of adherence to recommendations for clinical practice.

**Supplementary Information:**

The online version contains supplementary material available at 10.1186/s12890-024-03194-4.

## Background

Chronic obstructive pulmonary disease (COPD) is the sixth leading cause of death in the United States (US) [1]. COPD is often characterized by periods of intensified disease activity, known as exacerbations. Exacerbations are characterized by a worsening of respiratory symptoms, such as cough, phlegm production, and dyspnea, which normally last for more than 1 or 2 days [2]. As COPD exacerbations are associated with a worsening of the disease, they are often linked with increased morbidity, mortality, and high healthcare costs [2, 3].

Previous studies, including the ECLIPSE study and a more recent retrospective study from Germany, have concluded that most patients with COPD are not frequent exacerbators in the first years after diagnosis [4, 5]. COPD treatment strategies are tailored based on the severity of symptoms and the frequency and risk of exacerbations. Moderate exacerbations have generally been treated with oral systemic corticosteroids, bronchodilators, and antibiotics, while severe exacerbations require hospitalization [3, 6].

Maintenance therapy may aid in preventing exacerbations. In 2011, the Global Initiative for Chronic Obstructive Lung Disease (GOLD) report recommended the treatment of patients with COPD using a short-acting bronchodilator, long-acting bronchodilator, or inhaled corticosteroids (ICS) and a long-acting bronchodilator, based on severity of the disease [3]. In the 2024 GOLD report, recommendations for maintenance therapy advocated the use of single or dual long-acting bronchodilator combination (long-acting beta-agonist [LABA] + long-acting muscarinic antagonist [LAMA]). Patients with at least two moderate exacerbations or at least one exacerbation per year leading to hospitalization were recommended to be treated with LABA + LAMA, with a consideration to use triple therapy (LABA + LAMA + ICS) for those with elevated blood eosinophil counts (≥ 300 cells/μL) plus a history of moderate/severe exacerbations in the year prior. The use of LABA + ICS for COPD is not recommended in the 2023 guidelines; instead, triple therapy should be considered where indicated [7].

Previous real-world evidence analyses conducted in 2012, 2014, and 2023 have shown that 59-71% of patients are not prescribed maintenance pharmacological therapy (long-acting agents) at initial diagnosis, and ICS is often overprescribed [8–10], in contrast to current guideline-based recommendations [3, 7]. Prompt and effective management with single or dual bronchodilation is recommended after the initial diagnosis of COPD to optimize lung function and prevent exacerbations [7].

In the US, patients presenting with COPD are typically initially evaluated by primary care physicians and only a subset of patients is referred to specialist care [11]. Generally, most patients with COPD receive care from a primary care provider, and lack of awareness of guidelines may lead to a delay between COPD diagnosis in the primary care setting and progression/referral to secondary care [12, 13]. Although GOLD advocates that spirometry should be undertaken to establish the diagnosis of COPD [7], only 30-37% of patients are diagnosed with spirometry, with most cases being diagnosed clinically [11, 14–16].

The potential impact of delaying optimal treatment on exacerbations and care utilization remains unclear. Here, we report the results of a retrospective inception cohort study in which the frequency of COPD exacerbations was determined in newly diagnosed patients throughout their patient journey in the 4 years after diagnosis. We also report exacerbation outcomes for patients who did not receive guideline-based therapy after the initial diagnosis and the treatment patterns of pharmacological interventions in the 4 years of follow-up.

## Methods

### Study design

This retrospective, noninterventional study used de-identified data from the Inovalon administrative claims database to create an inception cohort. The study period was between January 1, 2015, and December 31, 2021.

The Inovalon database is representative of the insured US population and includes over 150 million covered lives between 2015 and 2021, including individuals insured with commercial, Medicaid Managed Care, and Medicare Advantage. The Inovalon Insights’ database provides information about patients across multiple plan types, offers the ability to follow patients longitudinally, and is compliant with the Health Insurance Portability and Accountability Act (HIPAA) regulations. Additionally, mortality data for the study population were obtained from Datavant; Datavant comprises aggregate mortality data that covers more than 85% of US deaths.

The study design incorporated a 1-year pre-index period before the index date to establish the baseline period and a 4-year follow-up period to characterize COPD exacerbations (Supplementary Fig. S1). The variables collected from the database included COPD exacerbations (moderate or severe), pharmacologic treatment patterns, demographic characteristics (age, sex, region, and insurance type), and the calendar year of cohort entry.

This study was conducted in compliance with the principles of the Declaration of Helsinki and the Guidelines for Good Pharmacoepidemiology Practice. The study was reviewed and approved by the Pearl Institutional Review Board (Indianapolis, IN, USA; 19-KANT-204).

### Study population

Patients were included in the study cohort if they had received a diagnosis of COPD between January 1, 2016, and December 31, 2017, defined as any inpatient or outpatient claim [17]. Furthermore, patients must have had continuous medical and prescription insurance enrollment for 12 months in the pre-index period up until 4 years post-index date or until death and be aged ≥ 40 years at the index COPD diagnosis date.

Patients were excluded if, during the pre-index period, they were diagnosed with asthma or other respiratory diseases, COPD, any cancer, or clinically significant cardiovascular disease. Patients were also excluded if they had a history of lung surgery in the pre-index period, history of severe alpha-1-antitrypsin deficiency during the pre-index period, evidence of any endobronchial valve placement procedure during the study period, evidence of moderate or severe exacerbation in the 4 weeks prior to the COPD diagnosis index date, repeated hospital visits or admissions for respiratory infections, or evidence of COPD exacerbation in the pre-index period without receiving a COPD diagnosis (12 months).

### Outcome measures

The primary outcome measure of the study was the number and rate of moderate and/or severe exacerbations, assessed using diagnostic codes for relevant diagnoses and the National Drug Code for prescription medications during the 4-year follow-up period.

Secondary outcome measures included sociodemographic characteristics, including age, sex, region, and insurance type, as well as clinical characteristics, including comorbidities and medication use. Treatment classes were determined based on the last claim prior to the specific 12-month block being assessed, including untreated (comprising those who received rescue medication [short-acting] or no COPD medications), LAMA or LABA monotherapy, LABA + ICS, LAMA + ICS, LAMA + LABA, or triple therapy (LABA + LAMA + ICS). The treatment patterns were determined at the time of COPD diagnosis and at the end of years 1, 2, 3, and 4.

### Definitions

Initial exacerbations were defined as the first exacerbations following the index COPD diagnosis. Documentation of more than one exacerbation within a 2-week time frame was considered a single exacerbation, with the highest-severity exacerbation taking precedence. Subsequent exacerbations were defined as those occurring after a 2-week period following the initial exacerbation.

Moderate COPD exacerbations were defined based on one of the following criteria: an ambulatory (office/outpatient) visit with a COPD diagnosis in any position + a pharmacy claim for an oral corticosteroid (OCS) prescription within ± 7 days of this visit, an ambulatory visit with a COPD diagnosis in any position + a pharmacy claim for an antibiotic within ± 7 days of this visit, or an ambulatory visit with a COPD diagnosis in any position + a pharmacy claim for an OCS and an antibiotic prescription within ± 7 days of this visit.

Severe COPD exacerbations were defined based on one of the following criteria: inpatient admission or emergency department (ED) visit with a COPD diagnosis in the primary position; inpatient admission or ED visit with an acute respiratory failure diagnosis in the primary position and a COPD diagnosis in any position; inpatient admission or ED visit with a pneumonia diagnosis in the primary position and a COPD diagnosis in any position; or inpatient admission or ED visit with an acute respiratory failure diagnosis plus an inpatient admission/ED visit within ± 7 days with a COPD diagnosis code in any position.

Untreated patients were defined as those who received short-acting rescue medication only (no maintenance COPD medication) or those receiving no treatment for COPD.

### Outcomes

The primary outcome of this study was the number and rate of moderate or severe exacerbations among patients with newly diagnosed COPD over a 4-year period (irrespective of treatment). Participants who died during a measurement period were excluded from that corresponding (current) period and all future periods.

Secondary outcomes included the evaluation of the cumulative incidence of moderate and severe exacerbations and the mean cumulative count of moderate and severe exacerbations among patients with newly diagnosed COPD in 12-month time periods over 4 years. Furthermore, we characterized the sociodemographic and clinical characteristics of patients who were treated and those who remained untreated at the time of diagnosis, assessed the prescribed treatment classes for (initially) untreated patients at year 1 (and at 12-month time periods for 4 years for each “untreated” patient group), and evaluated the rates of moderate and severe exacerbations in patients who remained untreated after diagnosis in 12-month time periods for 4 years.

In a separate analysis, the rate of moderate and severe exacerbations among the untreated patient population was also examined.

### Statistical analysis

The covariates included age, sex, region, insurance type, calendar year of cohort entry, prescriber specialty, comorbidities, healthcare utilization, and other medication use. De-identified, processed, and cleaned data were directly exported from Inovalon into the statistical software SAS 9.4 (SAS Institute, Cary, NC, US). Data were analyzed by Cerner Enviza.

Descriptive statistics were reported for all study variables, including baseline characteristics and outcomes. Frequencies and percentages were reported for categorical variables, with means and standard deviation (SD) or variable ranges for continuous or discrete variables. Moderate and severe exacerbations were assessed separately as well as in combination (any exacerbation). Among patients newly diagnosed with COPD, the proportion of patients with moderate and/or severe COPD exacerbations was calculated as follows:

[Number of patients with moderate/severe exacerbations] ÷ [Total cohort].

The rate of moderate and/or severe COPD exacerbations was also calculated in 12-month time periods among the overall population as well as among the untreated population as follows:

[Number of moderate/severe exacerbations] ÷ [Person-years of follow-up since COPD diagnosis over the entire 4-year follow-up period (in 12-month blocks)].

## Results

### Participants

A total of 238,158 patients with a COPD diagnosis met the eligibility criteria and were included in the cohort (Supplementary Fig. S2). The mean (SD) age of the cohort was 63.8 (22.2) years. The majority of patients were female (52.9% vs. 47.1% male), residing in the South (40.5% vs. 17.1% in the Northeast, 22.1% in the Midwest, and 20.3% in the West), insured by Medicaid Managed Care (46.2% vs. 29.1% by commercial, 37.3% by Medicare Advantage, and 6.5% by unknown insurance), and entered the cohort in 2016 (67.3% vs. 32.7% in 2017) (Table 1).

**Table 1:** Characteristics of the study population

Characteristics		Total (*N* = 238,158)
Age (Years)	Mean (SD)	63.8 (22.2)
Sex	Female	126,087 (52.9)
	Male	112,069 (47.1)
	Unknown	2 (0.0)
Region	Northeast	40,707 (17.1)
	Midwest	52,593 (22.1)
	South	96,442 (40.5)
	West	48,391 (20.3)
	Unknown	25 (0.0)
Insurance Type	Commercial	69,286 (29.1)
	Medicaid Managed Care	110,133 (46.2)
	Medicare Advantage	88,913 (37.3)
	Unknown	15,463 (6.5)
Cohort Entry Year	2016	160,353 (67.3)
	2017	77,805 (32.7)

Data are presented as n (%) unless specified otherwise

SD: standard deviation

### Exacerbations across clinical characteristics

Of the total cohort, 61,516 (25.8%) and 32,935 (13.8%) patients experienced moderate and severe exacerbations, respectively, during the 4-year follow-up period (Table 2). Overall, 32.9% of the patients had at least one exacerbation (moderate or severe), and of all exacerbations that occurred during follow-up, 69.1% were moderate and 30.9% were severe (Table 2).

**Table 2:** Proportions and mean number of moderate, severe, and any exacerbations, overall and by patient characteristics

Total (*N* = 238,158)		Moderate Exacerbation *n* = 61,516			Severe Exacerbation *n* = 32,935			Any Exacerbation *n* = 78,367		
		Unique Patients (% of the Total Cohort)	Overall Exacerbations (% of the Total Exacerbations)	Mean (SD) Number of Exacerbations	Unique Patients (% of the Total Cohort)	Overall Exacerbations (% of the Total Exacerbations)	Mean (SD) Number of Exacerbations	Unique Patients (% of the Total Cohort)	Overall Exacerbations (% of the Total Exacerbations)	Mean (SD) Number of Exacerbations
		61,516 (25.8)	128,794 (69.1)	-	32,935 (13.8)	57,581 (30.9)	-	78,367 (32.9)	186,375 (100)	-
Sex (Missing Sex = 2)	Female	35,086 (57.0)	76,072 (59.1)	2.17 (1.91)	18,603 (56.5)	32,288 (56.1)	1.74 (1.55)	44,208 (56.4)	108,360 (58.1)	2.45 (2.26)
	Male	26,430 (43.0)	52,722 (40.9)	1.99 (1.75)	14,332 (43.5)	25,293 (43.9)	1.76 (1.67)	34,159 (43.6)	78,015 (41.9)	2.28 (2.14)
Age Group (Years)	< 45	2,089 (3.4)	4,372 (3.4)	2.09 (1.88)	1,115 (3.4)	1,905 (3.3)	1.71 (1.46)	2,707 (3.5)	6,277 (3.4)	2.32 (2.12)
	45–54	13,761 (22.4)	29,446 (22.9)	2.14 (1.91)	7,616 (23.1)	13,876 (24.1)	1.82 (1.68)	17,633 (22.5)	43,322 (23.2)	2.46 (2.30)
	55–64	24,754 (40.2)	52,251 (40.6)	2.11 (1.88)	13,493 (41.0)	24,263 (42.1)	1.80 (1.71)	31,655 (40.4)	76,514 (41.1)	2.42 (2.29)
	65–74	14,077 (22.9)	29,376 (22.8)	2.09 (1.83)	6,871 (20.9)	11,588 (20.1)	1.69 (1.50)	17,491 (22.3)	40,964 (22.0)	2.34 (2.14)
	> 74	6,835 (11.1)	13,349 (10.4)	1.95 (1.58)	3,840 (11.7)	5,949 (10.3)	1.55 (1.22)	8,881 (11.3)	19,298 (10.4)	2.17 (1.86)
Region (Missing Location = 25)	Northeast	9,763 (15.9)	19,628 (15.2)	2.01 (1.74)	6,187 (18.8)	11,390 (19.8)	1.84 (1.81)	13,244 (16.9)	31,018 (16.6)	2.34 (2.22)
	Midwest	13,951 (22.7)	28,859 (22.4)	2.07 (1.80)	7,026 (21.3)	11,550 (20.1)	1.64 (1.42)	17,498 (22.3)	40,409 (21.7)	2.31 (2.09)
	South (includes PR)	27,326 (44.4)	59,401 (46.1)	2.17 (1.92)	13,746 (41.7)	23,912 (41.5)	1.74 (1.60)	33,903 (43.3)	83,313 (44.7)	2.46 (2.28)
	West	10,470 (17.4)	20,899 (16.2)	2.00 (1.81)	5,975 (18.1)	10,728 (18.8)	1.80 (1.60)	13,716 (17.5)	31,627 (17.0)	2.31 (2.16)
	Missing	6 (0.0)	7 (0.0)	1.17 (0.41)	1 (0.0)	1 (0.0)	1.00 (-)	6 (0.0)	8 (0.0)	1.33 (0.52)
Insurance Type	Commercial	8,963 (14.6)	18,671 (14.5)	2.08 (1.85)	3,202 (9.7)	4,706 (8.2)	1.47 (1.09)	10,432 (13.3)	23,377 (12.5)	2.24 (2.06)
	Medicaid Managed Care	19,723 (32.1)	41,620 (32.3)	2.11 (1.90)	13,719 (41.7)	25,857 (44.9)	1.88 (1.81)	27,060 (34.5)	67,477 (36.2)	2.49 (2.37)
	Medicare Advantage	14,740 (24.0)	30,322 (23.5)	2.06 (1.77)	7,503 (22.8)	12,459 (21.6)	1.66 (1.45)	18,661 (23.8)	42,781 (23.0)	2.29 (2.07)
	Unknown	18,090 (29.4)	38,181 (29.6)	2.11 (1.84)	8,511 (25.8)	14,559 (25.3)	1.71 (1.53)	22,214 (28.3)	52,740 (28.3)	2.37 (2.18)
Cohort Entry Year	2016	46,369 (75.4)	100,243 (77.8)	2.16 (1.90)	24,258 (73.7)	43,164 (75.0)	1.78 (1.64)	57,946 (73.9)	143,407 (76.9)	2.47 (2.29)
	2017	15,147 (24.6)	28,551 (22.2)	1.88 (1.65)	8,677 (26.3)	14,417 (25.0)	1.66 (1.49)	20,421 (26.1)	42,968 (23.1)	2.10 (1.95)

COPD: chronic obstructive pulmonary disease; PR: Puerto Rico; SD: standard deviation

Patients who had moderate and severe exacerbations were predominantly female (57.0% and 56.5%, respectively), aged 55–64 years (40.2% and 41.0%), residing in the South (44.4% and 41.7%), and insured by Medicaid Managed Care (32.1% and 41.7%). However, there were no clear differences, in terms of sex, age, region, or insurance type, between those with moderate or severe exacerbation types (Table 2). The mean number of moderate and severe exacerbations was similar across sex, age, region, and insurance type (Table 2). Furthermore, the mean number of moderate and severe exacerbations remained relatively stable over the 4-year follow-up period (Table 2).

Exacerbations occurred most frequently in the first year after COPD diagnosis and trended downward following the first year. In year 1, 43.2% of patients had a moderate exacerbation, which decreased to 30.8% in year 4 (Table 3). This pattern was also observed for severe exacerbations, with 41.8% of patients having a severe exacerbation in year 1 and 27.0% in year 4 (Table 3).

**Table 3:** Proportions and mean number of moderate, severe, and any exacerbations by year after COPD diagnosis

Total (*N* = 238,158)		Moderate Exacerbation *n* = 61,516			Severe Exacerbation *n* = 32,935			Any Exacerbation *n* = 78,367		
		Unique Patients (% of the Total Cohort)	Overall Exacerbations (% of the Total Exacerbations)	Mean (SD) Number of Exacerbations	Unique Patients (% of the Total Cohort)	Overall Exacerbations (% of the Total Exacerbations)	Mean (SD) Number of Exacerbations	Unique Patients (% of the Total Cohort)	Overall Exacerbations (% of the Total Exacerbations)	Mean (SD) Number of Exacerbations
		61,516 (25.8)	128,794 (69.1)	-	32,935 (13.8)	57,581 (30.9)	-	78,367 (32.9)	186,375 (100)	-
Exacerbations by Year After the COPD Index Date^*^	Year 1	26,594 (43.2)	35,070 (27.2)	1.32 (0.70)	13,762 (41.8)	17,410 (30.2)	1.27 (0.69)	37,430 (38.0)	52,480 (23.2)	1.40 (0.82)
	Year 2	25,768 (41.9)	35,046 (27.2)	1.36 (0.76)	11,539 (35.0)	14,906 (25.9)	1.29 (0.74)	34,383 (34.9)	49,952 (22.1)	1.45 (0.89)
	Year 3	23,925 (38.9)	33,005 (25.6)	1.38 (0.79)	10,649 (32.3)	13,783 (23.9)	1.29 (0.76)	31,721 (32.2)	46,788 (20.7)	1.47 (0.93)
	Year 4	18,939 (30.8)	25,673 (19.9)	1.36 (0.76)	8,898 (27.0)	11,482 (19.9)	1.29 (0.73)	25,732 (26.1)	37,155 (16.5)	1.44 (0.89)

COPD: chronic obstructive pulmonary disease; SD: standard deviation. *Patients could have had more than one exacerbation

*Rate of moderate*,* severe*,* and any exacerbations (in person-years) among newly diagnosed patients over the 4-year study period*.

**Fig. 1:**
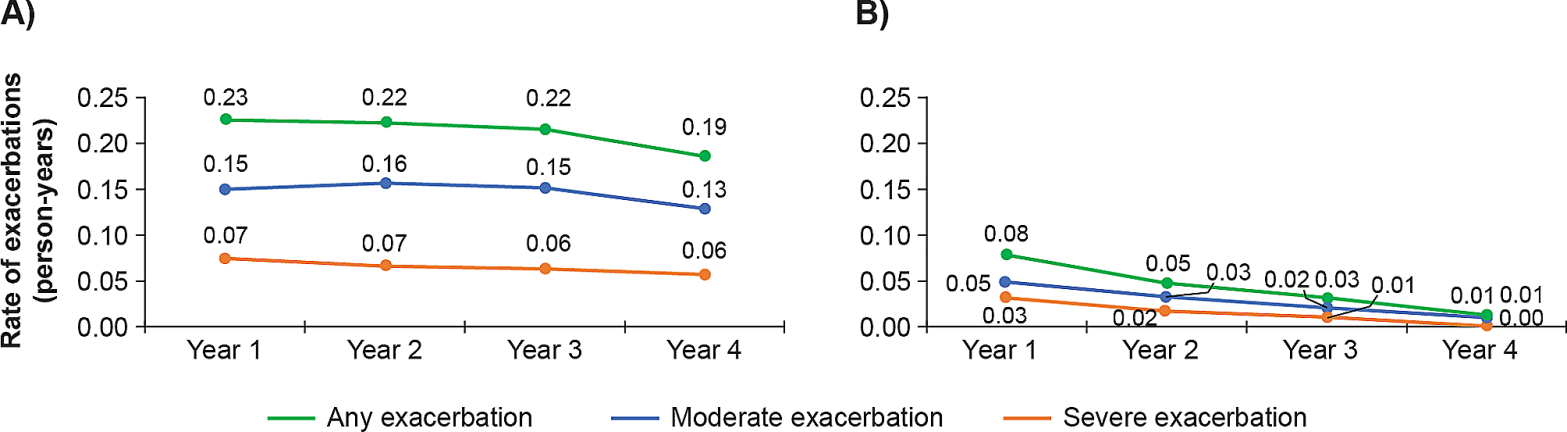
Exacerbation rate (in person-years) among patients newly diagnosed with COPD over the 4-year study period. (**A**) Exacerbation rate after censoring for mortality^*^; (**B**) Exacerbation rate after censoring for mortality and treatment† *Censoring for mortality: Patients who died during each 12-month period were excluded from the corresponding period and all future periods †Censoring for mortality and treatment: In addition to censoring for mortality, patients who initiated treatment during a 12-month period were excluded from that and future measurement periods COPD: chronic obstructive pulmonary disease

After exclusion for mortality (Supplementary Table S1), the rate of exacerbations peaked in the first year, after which it remained fairly constant over the 4-year period (Fig. 1A). The rates of moderate and severe exacerbations in the first year following the initial COPD diagnosis were 0.15/person-years and 0.07/person-years, respectively (Fig. 1A). The rates of moderate and severe exacerbations, respectively, in the subsequent years were 0.16/person-years and 0.07/person-years for year 2, 0.15/person-years and 0.06/person-years for year 3, and 0.13/person-years and 0.06/person-years for year 4 (Fig. 1A).

After exclusion for maintenance treatment in addition to mortality, exacerbation rates peaked at year 1 but were relatively low across the entire 4-year period following the initial COPD diagnosis, and the rates declined gradually over the follow-up period (Fig. 1B). In the initial year following COPD diagnosis, the rate of moderate exacerbations was 0.05/person-years and that of severe exacerbations was 0.03/person-years (Fig. 1B). The rates of moderate and severe exacerbations after COPD diagnosis, respectively, were 0.03/person-years and 0.02/person-years in year 2, 0.02/person-years and 0.01/person-years in year 3, and 0.01/person-years and 0.00/person-years in year 4 (Fig. 1B).

### Subgroup analyses

We compared the rates of exacerbations by the place of diagnosis and found that the rates of both moderate and severe exacerbations were higher among those diagnosed in the inpatient setting (*N* = 90,678) than those diagnosed in the outpatient setting (*N* = 147,480), and this was consistent across the entire 4-year period (Supplementary Table S2, Supplementary Fig. S3). In both inpatient and outpatient settings, the rates of moderate and severe exacerbations peaked in the first year of diagnosis and then gradually declined (Supplementary Table S2, Supplementary Fig. S3).

### Demographics of the treatment groups

In the overall cohort (*N* = 238,158), 32,981 (13.8%) patients were treated within 30 days of being diagnosed with COPD, 53,187 (22.3%) patients received treatment during follow-up after diagnosis, and 151,990 (63.8%) patients remained untreated during the entire 4-year follow-up period (Table 4). The sex distribution was similar across all treatment groups (Table 4). Patients aged 55–64 years constituted the highest proportion across all treatment groups (Table 4). Medicaid was the most common insurance type across all treatment categories (Table 4).

**Table 4:** Demographics and clinical characteristics across treatment groups

		Treated With Maintenance Therapy at Diagnosis (*n* = 32,981)	Treated With Maintenance Therapy^†^ After Diagnosis (*n* = 53,187)	Untreated During the 4-Year Follow-up‡ (*n* = 151,990)	
		n (%)	n (%)	n (%)	
Sex (missing = 2)	Female	17,869 (54.2)	29,680 (55.8)	78,538 (51.7)	
	Male	15,112 (45.8)	23,507 (44.2)	73,450 (48.3)	
Age Group (years)	< 45	1,242 (3.8)	1,909 (3.6)	6,000 (3.9)	
	45–54	7,931 (24.0)	12,292 (23.1)	28,793 (18.9)	
	55–64	14,445 (43.8)	22,335 (42.0)	49,467 (32.5)	
	65–74	6,290 (19.1)	11,395 (21.4)	41,402 (27.2)	
	> 74	3,073 (9.3)	5,256 (9.9)	26,328 (17.3)	
Region	Northeast	6,621 (20.1)	11,061 (20.8)	23,025 (15.1)	
	Midwest	7,982 (24.2)	11,503 (21.6)	33,108 (21.8)	
	South	13,478 (40.9)	21,833 (41.0)	61,131 (40.2)	
	West	4,896 (14.8)	8,785 (16.5)	34,710 (22.8)	
	Unknown	4 (0.0)	5 (0.0)	16 (0.0)	
Insurance Type	Commercial	4,859 (14.7)	7,930 (14.9)	28,481 (18.7)	
	Medicaid Managed Care	11,792 (35.8)	18,686 (35.1)	45,458 (29.9)	
	Medicare Advantage	6,793 (20.6)	12,873 (24.2)	31,354 (20.6)	
	Unknown/Other	9,537 (28.9)	13,698 (25.8)	46,697 (30.7)	
Cohort Entry Year	2016	24,022 (72.8)	37,653 (70.8)	98,678 (64.9)	
	2017	8,959 (27.2)	15,534 (29.2)	53,312 (35.1)	
Number of Patients with Exacerbations by Treatment^*^	Moderate	14,734 (44.7)	24,730 (46.5)	22,052 (14.5)	
	Severe	7,873 (23.9)	14,122 (26.6)	10,940 (7.2)	
	Any	17,961 (54.5)	30,967 (58.2)	29,439 (19.4)	

COPD: chronic obstructive pulmonary disease; SD: standard deviation. *Patients could have had more than one exacerbation; ^†^With/Without Short-Acting Medications; ^‡^Could Have Received Short-Acting Medications

Exacerbations were more common among those who were treated than those who remained untreated, with 44.7% and 23.9% of those treated at diagnosis with moderate and severe exacerbations, respectively (Table 4). Likewise, 46.5% and 26.6% of the patients treated during the follow-up period had moderate and severe exacerbations, respectively (Table 4).

### Treatment patterns across therapy classes

At the time of diagnosis, the majority (*n* = 205,177; 86.2%) of patients were untreated. Given that 40.4% (*n* = 96,258) of patients were prescribed short-acting bronchodilators at diagnosis, we assessed the time until escalation to maintenance therapy within the 4 years of follow-up. Out of the group who received a short-acting prescription at diagnosis, 15.4% (36,610) of patients received a long-acting prescription during follow-up, and 25.0% (*n* = 59,648) remained on short-acting treatment. The median time to first long-acting treatment prescription among patients prescribed a short-acting treatment initially was 6.0 months (interquartile range [IQR] 1.0–19.0). In this group, LABA + ICS was prescribed in 61.2%, LAMA monotherapy in 23.5%, LAMA + LABA in 12.0%, LABA in 1.7%, and triple therapy in 1.6% of patients.

By the end of year 1, 13.2% of those who were untreated at diagnosis (*n* = 205,177) had received some form of treatment (63.7% received a LABA + ICS, 25.5% received LAMA monotherapy, 9.1% received LAMA + LABA, 1.5% received LABA monotherapy, and 0.1% were prescribed a triple therapy), and by the end of year 4, 3.7% of those who were previously untreated (*n* = 157,896) had received treatment (58.8% received a LABA + ICS, 18.6% received LAMA monotherapy, 15.9% received LAMA + LABA, 5.6% were prescribed a triple therapy, and 1.1% received LABA monotherapy) (Table 5).

**Table 5:** Patterns of first treatment across therapy classes, followed in 12-month blocks after COPD diagnosis

		At the Time of Diagnosis Treated *n* = 32,981		At the End of Year 1 Treated *n* = 27,141		At the End of Year 2 Treated *n* = 11,865		At the End of Year 3 Treated *n* = 8,275		At the End of Year 4 Treated *n* = 5,906	
		Patients (n)	% Among Treated	Patients (n)	% Among Treated	Patients (n)	% Among Treated	Patients (n)	% Among Treated	Patients (n)	% Among Treated
Group	LABA Monotherapy	415	1.3	416	1.5	193	1.6	127	1.5	66	1.1
	LAMA Monotherapy	9,983	30.3	6,929	25.5	2,666	22.5	1,637	19.8	1,098	18.6
	LABA + ICS	20,459	62.0	17,287	63.7	7,164	60.4	4,864	58.8	3,475	58.8
	LAMA + LABA	2,123	6.4	2,473	9.1	1,719	14.5	1,360	16.4	937	15.9
	LAMA + LABA + ICS	1	0.0	36	0.1	123	1.0	287	3.5	330	5.6
	Untreated	205,177		178,036		166,171		157,896		151,990	

COPD: chronic obstructive pulmonary disease; ICS: inhaled corticosteroids; LABA: long-acting beta-agonist; LAMA: long-acting muscarinic antagonist

**Fig. 2:**
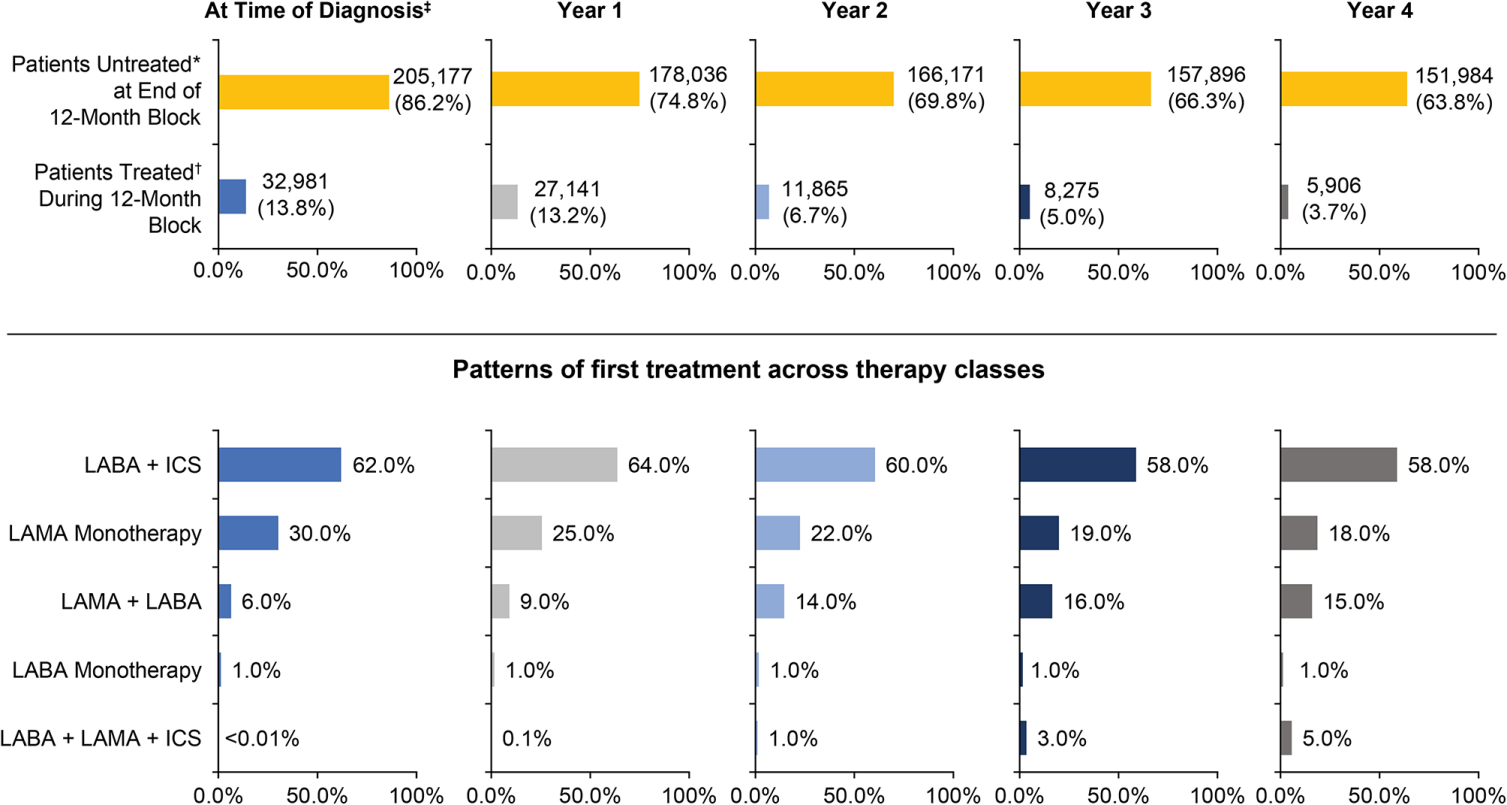
Patterns of first maintenance treatment across therapy classes among treated patients with COPD Treatment percentages represent the total proportion of newly treated patients prescribed a particular treatment therapy class in a 12-month period. For example, a total of 27,141 patients were treated in year 1, 62.78% of whom were treated with LABA + ICS, while in year 2, 60.38% of the total treated patients (*n* = 11,865) received LABA + ICS therapy *Untreated patients with COPD were defined as those who received rescue medication only and no maintenance COPD medication †Treated patients with COPD were defined as those with ≥ 1 prescription claim(s) for LAMA and/or LABA; LABA and ICS; LAMA and ICS; or LAMA, LABA, and ICS. ‡Defined as the 30-day post-index period (to accommodate delayed prescription filling time) COPD: chronic obstructive pulmonary disease; ICS: inhaled corticosteroids; LABA: long-acting beta-agonist; LAMA: long-acting muscarinic antagonist

At the time of diagnosis, the most commonly received therapy among those receiving maintenance therapy was LABA + ICS (62.0%), which remained the most common form of treatment at the end of years 1 (64.0%), 2 (60.0%), 3 (58.0%), and 4 (58.0%) (Fig. 2). Triple therapy was the least commonly received treatment, but its use increased over the years from < 0.01% at diagnosis to 5.0% at year 4 of follow-up (Fig. 2). Of the total cohort, most patients (38.4%) received short-acting beta agonists (SABAs) at the end of year 1, which remained the most common treatment throughout the 4-year follow-up period (Table 6). The second and third most common treatments were LABA + ICS and ICS alone during the entire 4-year follow-up period, respectively (Table 6).

**Table 6:** Treatment patterns among overall cohort of patients with COPD

		Year 1			Year 2		Year 3		Year 4	
		Patients (n, Not Unique)	% of Total Cohort	% of All Year 1 Claims	Patients (n, Not Unique)	% of All Year 2 Claims	Patients (n, Not Unique)	% of All Year 3 Claims	Patients (n, Not Unique)	% of All Year 4 Claims
Group	ICS	39,093	16.4	14.7	34,826	14.5	32,751	13.8	28,977	13.2
	LABA Monotherapy	1,218	0.5	0.4	1,035	0.5	1,882	0.7	3,968	1.4
	LAMA Monotherapy	23,661	9.9	12.1	19,905	11.8	18,063	11.2	16,295	10.5
	LABA + ICS	43,667	18.3	21.4	38,299	21.9	36,230	21.4	33,518	20.5
	LAMA + LABA	5,941	2.5	2.4	6,863	3.4	7,759	4.2	7,842	4.6
	LABA + LAMA+ ICS	103	0.0	0.0	702	0.2	2,387	1.0	4,022	2.1
	Methylxanthine Monotherapy	927	0.4	0.5	833	0.5	714	0.5	614	0.4
	PDE Monotherapy	472	0.2	0.3	495	0.3	558	0.3	622	0.4
	SABA Monotherapy	91,418	38.4	39.6	75,805	38.6	71,640	38.7	65,606	39.1
	SAMA Monotherapy	6,838	2.9	2.4	5,720	2.3	5,324	2.2	4,676	2.0
	SAMA + SABA	17,031	7.2	6.1	13,869	6.0	13,705	6.0	12,259	5.8

COPD: chronic obstructive pulmonary disease; ICS: inhaled corticosteroids; LABA: long-acting beta-agonist; LAMA: long-acting muscarinic antagonist; PDE: phosphodiesterase; SABA: short-acting beta-agonist; SAMA: short-acting muscarinic antagonist

## Discussion

In this retrospective study, we established an inception cohort of US patients newly diagnosed with COPD and followed their patient journey for 4 years after diagnosis. We found that 25.8% and 13.8% of the patients diagnosed with COPD (*N* = 238,158) had moderate and severe exacerbations, respectively. Additionally, 32.9% of the cohort had at least one moderate or severe exacerbation during the entire 4-year follow-up period.

Over the 4-year follow-up period, 63.8% of patients did not receive long-acting maintenance treatment, which is recommended by current guidelines to help optimize lung function, reduce exacerbations, and improve quality of life [7]. These exacerbation rates were slightly lower than those reported previously [18]. Pasquale et al. performed a retrospective analysis of pharmacy claims data obtained between 2007 and 2009 and found that in the 2 years of follow-up after diagnosis, 49.8% of the overall population had an exacerbation [18]. The study focused on patients with COPD who had chronic bronchitis, which is different from the population in our study.

Although the proportion of exacerbations was lower than that observed in previous studies, those who had exacerbations had multiple exacerbations, 25.8% (*n* = 61,516) had at least one moderate exacerbation, which contributed to 69.1% (*n* = 128,794) of total exacerbations; similarly, 13.8% (*n* = 32,935) of severe exacerbators contributed to 30.9% (*n* = 57,581) of overall exacerbations. Exacerbation rates were noticeably lower among those who remained untreated during each year of follow-up and exacerbation rates among all patients decreased with each additional year of follow-up (exacerbation [any] rate in person-years for all patients newly diagnosed with COPD versus those newly diagnosed with COPD and were untreated: 0.23 vs. 0.08; 0.22 vs. 0.05; 0.22 vs. 0.03; 0.19 vs. 0.01, for years 1, 2, 3, and 4, respectively).

The exacerbation rates in this study may reflect an incident COPD population with a relatively low disease severity. A study conducted in Germany by Vogelmeier et al. grouped patients according to their baseline exacerbation risk as follows: (1) patients with no exacerbations in the 12 months prior, (2) patients with one moderate prior exacerbation, and (3) patients with either one severe or multiple prior exacerbations [5]. After 3 years of follow-up, the Vogelmeier study reported that most patients in the latter two groups had exacerbations (59.1% and 81.7%, respectively), while 35.9% of patients in the first group (no exacerbations) experienced an exacerbation. The latter number is similar to our findings (with 25.8% of patients having moderate and 13.8% severe exacerbations), suggesting that our cohort may consist of a less severe patient population who had no prior exacerbations (as we excluded patients with evidence of moderate or severe exacerbations in the 4 weeks prior to the COPD diagnosis index date and those with evidence of COPD exacerbations in the 12-month pre-index period).

We found that in patients who received maintenance treatment at baseline, the rate of exacerbations was 54.5% in year 1, suggesting that the baseline risk of exacerbations may be different across treatment categories, with those being treated for moderate-to-severe COPD at a higher risk of exacerbations due to the greater severity of the disease. This was confirmed when we assessed the number of exacerbations for patients diagnosed in the inpatient or outpatient settings, with those diagnosed in the inpatient setting having higher rates of exacerbations than those in the outpatient setting. While we were not able to directly examine the severity of COPD given the lack of clinical data (e.g., spirometry results) in claims, those diagnosed in the inpatient setting are likely to have more severe COPD, which could explain the higher rates of COPD exacerbation observed in this subgroup.

The exacerbation rates were fairly stable in the overall population (prior to treatment censoring) and consistent with those reported in a previous study, which suggested that the frequency of exacerbations does not increase over time [19]. In contrast, a study from Canada conducted in 2007 showed that those who experienced exacerbations were at a significantly higher risk of subsequent exacerbations [20]. The stable exacerbation rates in our study may be reflective of the study population, which comprised patients with their first diagnosis of COPD (of which a majority were diagnosed in the outpatient setting). Although patients diagnosed with asthma and other similar respiratory conditions were excluded, it is likely that most of our population may have had early/mild COPD. Our study included the period during the coronavirus disease 2019 (COVID-19) pandemic. This may have also affected the data, as patients were more home bound, were potentially more compliant with medication regimens, and may have practiced more frequent handwashing, which may collectively have resulted in less exposure to potential exacerbation triggers. After censoring at the initial treatment, our finding that the rates of exacerbation were high in the first year of follow-up and decreased in each 12-month period over time may be reflective of heightened health awareness and healthcare resource utilization in the period initially following diagnosis.

At the initial COPD diagnosis, 86.2% of patients with COPD were untreated (no long-acting therapies, no short-acting reliever medication, or no treatment), and by the end of the 4-year follow-up period, the majority (63.8%) remained untreated. This is consistent with a previous report from the US in 2012, which mentioned that 70.9% of patients in the Medicare population and 66.3% of those with commercial insurance did not receive maintenance treatment [8].

For those who did receive treatment, short-acting therapies, specifically SABAs (38.4% in year 1), were the most common treatment regimens for the patients in our cohort. Among the maintenance therapies prescribed, ICS + LABA was the most common therapy, followed by LAMA monotherapy and LAMA + LABA, which remained consistent across all years of follow-up. While ICS + LABA was a more common treatment during the time frame of this study, current guidelines suggest that this treatment is not optimal for most patients and that the LAMA + LABA combination is the preferred approach, offering advantages in terms of efficacy and tolerability [7]. The higher frequency of SABA use further suggests that a large proportion of our study population might have mild/early COPD.

The recommended guidelines for COPD treatment are LABA or LAMA used alone or in combination with the aim of relieving symptoms and improving quality of life [7]. Our finding that less than a third and only about a tenth of those treated received LAMA monotherapy and LAMA + LABA, respectively, suggests a lack of compliance with recommended guidelines and an opportunity to further improve patient care [7]. Understanding the reasons behind the noted prescribing patterns in this study would be essential for improving the quality of life and overall patient journey of patients newly diagnosed with COPD.

As with all real-world evidence studies, this data analysis has some limitations. As this study utilized claims data, most measures were based on healthcare utilization; this may have introduced a selection bias, as patients had to seek care in a healthcare setting to be included in our cohort. There is also a risk of misclassification bias related to the definition of our variables. To address this bias, we varied the definition of severe exacerbations. Furthermore, we could not include a measure of COPD severity because of the unavailability of clinical measures.

Despite these limitations, this is one of the first studies to provide evidence of exacerbations in patients with untreated COPD. This study included a 4-year longitudinal follow-up of patients with COPD in the US. When a sensitivity analysis was completed using the years pre-COVID-19 pandemic only, we noted a similar pattern in healthcare resource utilization (HCRU); however, the overall magnitude of HCRU was higher prior to COVID-19. To assess the robustness of our findings, sensitivity analyses were incorporated around COVID-19 periods, severe COPD exacerbations, and treatment definitions.

## Conclusions

This study highlights the significant undertreatment of COPD in commercial, Medicare Advantage, and Medicaid Managed Care patients with COPD. Most patients with COPD were not treated at the initial diagnosis and remained untreated by the end of the 4-year follow-up period. The exacerbation rates among patients newly diagnosed with COPD were low. Although we were not able to define disease severity for this cohort of patients, and they may have less severe disease, our results reflect previous studies that illustrate that the majority of patients are not frequent exacerbators. In addition, it is also possible (despite the requirement of two International Classification of Diseases [ICD] codes for COPD) that a small number of patients received incorrect diagnostic codes and did not actually have COPD. The recommended treatment guidelines were not adhered to in clinical practice, suggesting a lost opportunity to improve lung function, prevent exacerbations, and improve the quality of life in patients with newly diagnosed COPD.

### Electronic supplementary material

Below is the link to the electronic supplementary material.

Supplementary Material 1

## Data Availability

The data that support the findings of this study are available from Cerner Enviza, but restrictions apply to the availability of these data, which were used under license for the current study, and so are not publicly available. Data are however available from the authors upon reasonable request and with permission of Cerner Enviza.

## Abbreviations

COPD: Chronic obstructive pulmonary disease
COVID-19: Coronavirus disease 2019
ED: Emergency department
GOLD: Global Initiative for Chronic Obstructive Lung Disease
HCRU: Healthcare resource utilization
HIPAA: Health Insurance Portability and Accountability Act
ICD-10: International Classification of Diseases, 10th revision
ICS: Inhaled corticosteroids
IQR: Interquartile range
LABA: Long-acting beta-agonist
LAMA: Long-acting muscarinic antagonist
OCS: Oral corticosteroid
PDE: Phosphodiesterase
PR: Puerto Rico
SABA: Short-acting beta-agonist
SAMA: Short-acting muscarinic antagonist
SD: Standard deviation
US: United States
