# Supplementary material for: Treatment patterns in patients with newly diagnosed COPD in the USA
Source: BMC Pulm Med. 2024 Aug 17;24:395. doi: 10.1186/s12890-024-03194-4 (PMC11330015; doi:10.1186/s12890-024-03194-4)

**ADDITIONAL FILE**

**Supplementary Table S1:** Mortality across the 4-year study period

| **Follow-up year** | **Total Patients (*n*)** | **Mortality**  **(*n*)** | **% of Overall Cohort** |
| --- | --- | --- | --- |
| Year 1 | 238,158 | 9,903 | 4.16 |
| Year 2 | 228,255 | 7,661 | 3.22 |
| Year 3 | 220,594 | 5,960 | 2.50 |
| Year 4 | 214,634 | 4,356 | 1.83 |

**Supplementary Table S2**: Subgroup analysis - exacerbation rate across place of diagnosis, censored by mortality and treatment

|  | | Total  Person-Years in 12-Month Blocks - Censored by Mortality and Treatment | Exacerbation Rates | | | | | | | |
| --- | --- | --- | --- | --- | --- | --- | --- | --- | --- | --- |
|  |  |  | **Moderate** | | **Severe** | | **Severe (Prior Definition)** | | **Any** | |
|  |  |  | ***N*** | **Rates** | ***N*** | **Rates** | ***N*** | **Rates** | ***N*** | **Rates** |
|  |  |  |  |  |  |  |  |  |  |  |
| Inpatient Diagnosis (*n*=90,678) | Year 1 | 65,705.9 | 4,486 | 0.07 | 4,744 | 0.07 | 13,127 | 0.20 | 9,230 | 0.14 |
|  | Year 2 | 53,353.7 | 2,555 | 0.05 | 2,159 | 0.04 | 5,957 | 0.11 | 4,714 | 0.09 |
|  | Year 3 | 45,900.7 | 1,427 | 0.03 | 1,203 | 0.03 | 3,446 | 0.08 | 2,630 | 0.06 |
|  | Year 4 | 38,428.8 | 482 | 0.01 | 464 | 0.01 | 3,446 | 0.09 | 946 | 0.02 |
| Non-Inpatient Diagnosis  (*n*=147,480) | Year 1 | 119,439.1 | 4,300 | 0.04 | 690 | 0.01 | 1,454 | 0.01 | 4,990 | 0.04 |
|  | Year 2 | 106,941.6 | 2,332 | 0.02 | 374 | 0.00 | 775 | 0.01 | 2,706 | 0.03 |
|  | Year 3 | 99,700.8 | 1,397 | 0.01 | 190 | 0.00 | 411 | 0.00 | 1,587 | 0.02 |
|  | Year 4 | 89,076.3 | 540 | 0.01 | 64 | 0.00 | 411 | 0.00 | 604 | 0.01 |

**Supplementary Figure S1**: Study design

**
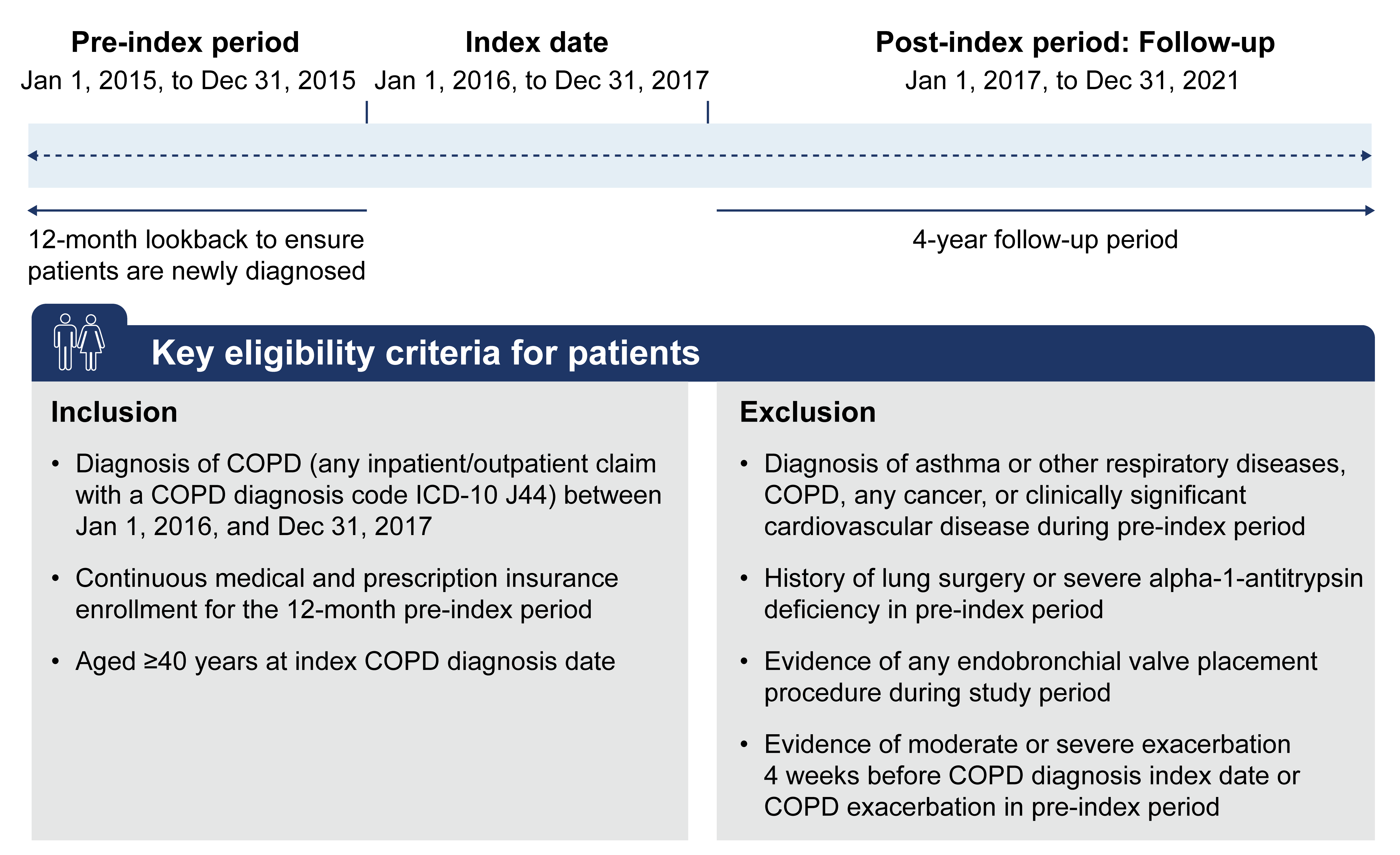
**

C*OPD: chronic obstructive pulmonary disease; ICD-10: International Statistical Classification of Diseases, 10^th^ revision.*

**Supplementary Figure S2:** Patient disposition

**
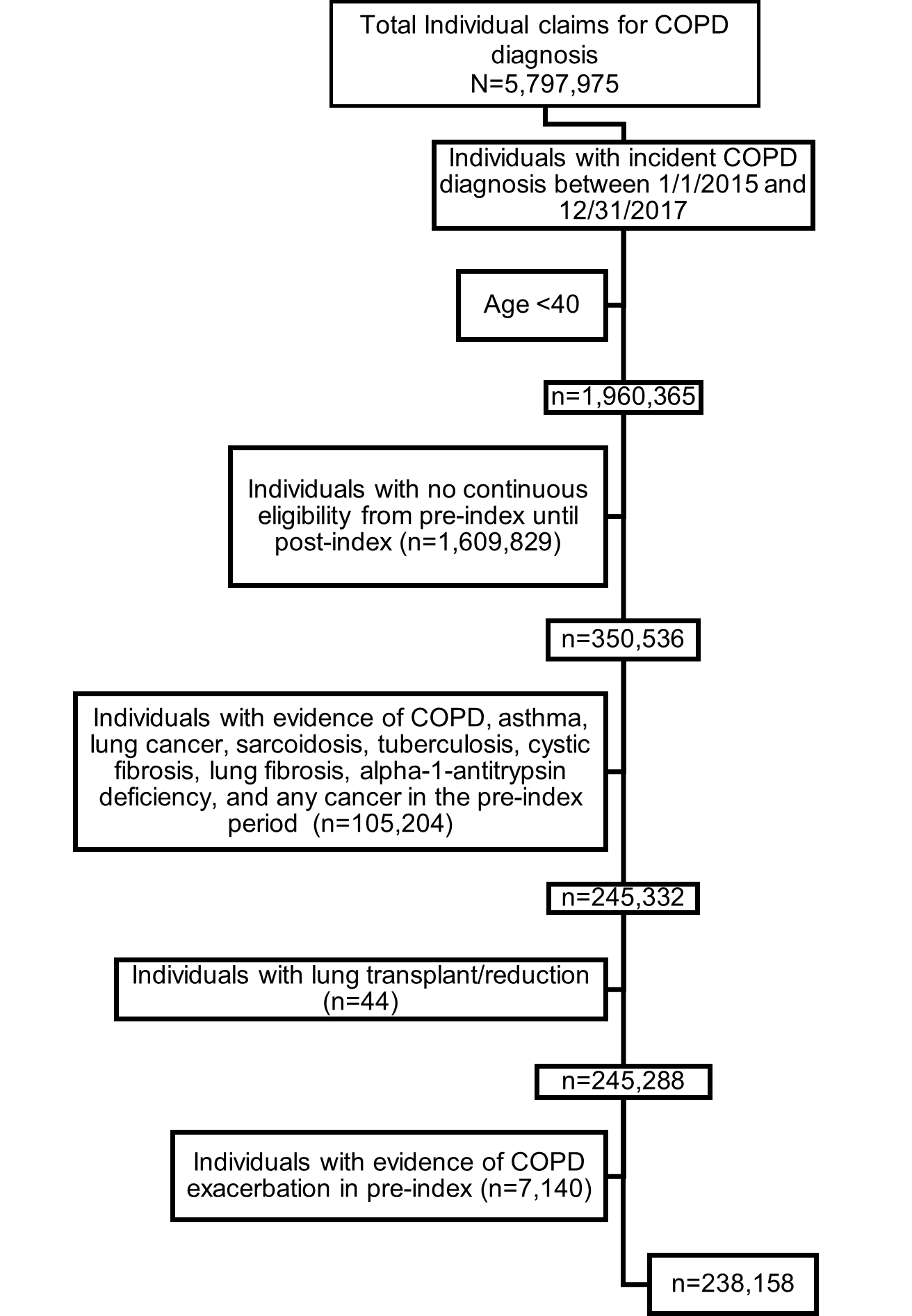
**

C*OPD: chronic obstructive pulmonary disease.*

**Supplementary Figure S3:** Subgroup analysis - exacerbation rate across diagnosis place of service, censored by mortality and treatment. A) Rate of moderate exacerbations; B) Rate of severe exacerbations; C) Rate of any exacerbations (moderate or severe).


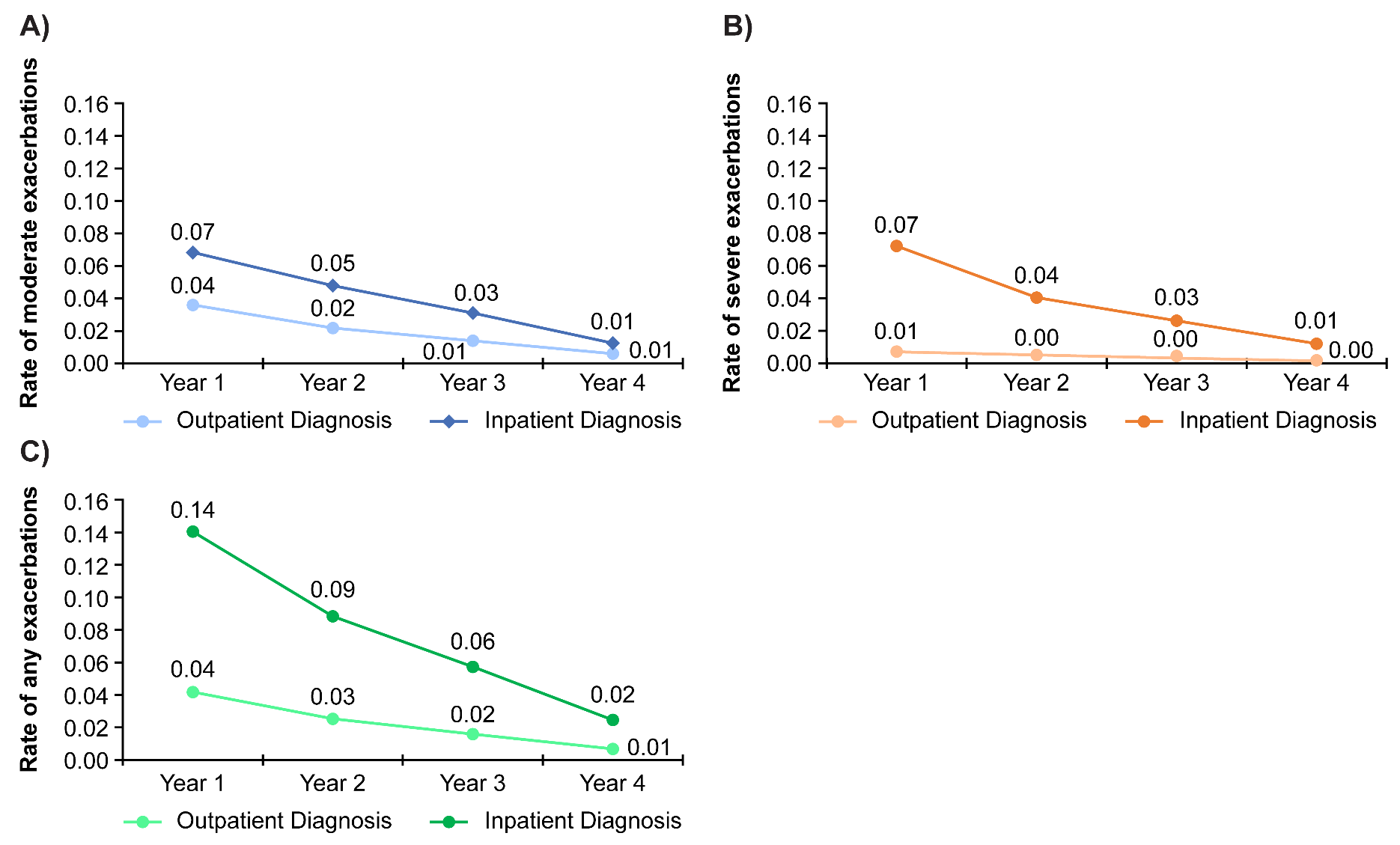

Supplement: Supplementary file 1 — Supplementary Material 1 [file 12890_2024_3194_MOESM1_ESM.docx]
